# A new spatiotemporal two-stage standardized weighted procedure for regional drought analysis

**DOI:** 10.7717/peerj.13249

**Published:** 2022-05-02

**Authors:** Rizwan Niaz, Nouman Iqbal, Nadhir Al-Ansari, Ijaz Hussain, Elsayed Elsherbini Elashkar, Sadaf Shamshoddin Soudagar, Showkat Hussain Gani, Alaa Mohamd Shoukry, Saad Sh. Sammen

**Affiliations:** 1 Statistics, Quaid-i-Azam University, Islamabad, Punjab, Pakistan; 2 Knowledge unit of business Economics accountancy and Commerce (KUBEAC), University of management and technology Sialkot campus, Sialkot, Pakistan; 3 Department of Civil, Environmental and Natural Resources Engineering, Lulea University of Technology, Lulea, Sweden; 4 Administrative Sciences Department, Community College, Riyadh, Riyadh, Saudi Arabia; 5 College of Business Administration, King Saud University Riyadh, Riyadh, Saudi Arabia, Riyadh, Saudi Arabia; 6 Business Administration, College of Business Administration, King Saud University Riyadh, Saudi Arabia, Riyadh, Riyadh, Saudi Arabia; 7 Arriyadh Community College, King Saud University, Riyadh, Saudi Arabia; 8 Workers University, KSA, Nsar, Egypt, Egypt; 9 Department of Civil Engineering, Coolege of Engineering, University of Diyala, Diyala Governorate, Iraq

**Keywords:** Spatiotemporal, Steady-state probabilities, Homogenous region, Meteorological stations, Standardized weighting scheme, Drought monitoring

## Abstract

Drought is a complex phenomenon that occurs due to insufficient precipitation. It does not have immediate effects, but sustained drought can affect the hydrological, agriculture, economic sectors of the country. Therefore, there is a need for efficient methods and techniques that properly determine drought and its effects. Considering the significance and importance of drought monitoring methodologies, a new drought assessment procedure is proposed in the current study, known as the Maximum Spatio-Temporal Two-Stage Standardized Weighted Index (MSTTSSWI). The proposed MSTTSSWI is based on the weighting scheme, known as the Spatio-Temporal Two-Stage Standardized Weighting Scheme (STTSSWS). The potential of the weighting scheme is based on the Standardized Precipitation Index (SPI), Standardized Precipitation Evapotranspiration Index (SPEI), and the steady-state probabilities. Further, the STTSSWS computes spatiotemporal weights in two stages for various drought categories and stations. In the first stage of the STTSSWS, the SPI, SPEI, and the steady-state probabilities are calculated for each station at a 1-month time scale to assign weights for varying drought categories. However, in the second stage, these weights are further propagated based on spatiotemporal characteristics to obtain new weights for the various drought categories in the selected region. The STTSSWS is applied to the six meteorological stations of the Northern area, Pakistan. Moreover, the spatiotemporal weights obtained from STTSSWS are used to calculate MSTTSSWI for regional drought characterization. The MSTTSSWI may accurately provide regional spatiotemporal characteristics for the drought in the selected region and motivates researchers and policymakers to use the more comprehensive and accurate spatiotemporal characterization of drought in the selected region.

## Introduction

Drought is a creeping phenomenon, influencing more individuals than other natural hazards (Botai et al., 2017; Mousavikhah, Shayegh & Ahmadpari, 2020; Bhunia, Das & Maiti, 2020; Elhoussaoui, Zaagane & Benaabidate, 2021). It is a slowly evolving and multifaceted disaster, often poorly understood in the perspective of regional climatic, hydrological, and human environment (Mukherjee, Mishra & Trenberth, 2018; Vicente-Serrano et al., 2020). Drought is a recurring natural hazard appearing in all climatic zones worldwide and significantly influences social and economic well-being, ecological environment, and agricultural sectors (Guneralp, Guneralp & Liu, 2015; Hagenlocher et al., 2019; Shah & Mishra, 2020). In its explicit form, drought can be described as the water discrepancy that appears in several types, including agricultural, meteorological, hydrological, and socio-economic drought (Lai et al., 2019; Jiang & Wang, 2019; Vernieuwe, De Baets & Verhoest, 2020; Diaz et al., 2020a). The meteorological drought occurs due to insufficient precipitation, and insufficiency in soil water supply triggers the agricultural drought. If drought distribution further continues *via* the hydrological cycle, a deficiency in surface or groundwater evolves, causing hydrological drought (Vernieuwe, De Baets & Verhoest, 2020; Diaz et al., 2020b).

Further, the monitoring, modeling, and prediction of meteorological droughts are of utmost significance (Mazhar et al., 2020; Guneralp, Guneralp & Liu, 2015; Hagenlocher et al., 2019; Jiang & Wang, 2019). Because the meteorological drought becomes the root for other drought types due to insufficient precipitation (Wu et al., 2017; Lai et al., 2019; West, Quinn & Horswell, 2019; Vernieuwe, De Baets & Verhoest, 2020). Moreover, accurate evaluation of meteorological drought brings useful information for decision-makers worldwide working in several fields associated with agriculture, hydrology, industrial, and water-budget managers to formulate precautionary measures and develop future planning (Alizadeh & Nikoo, 2018). Further, various studies have described the importance of monitoring regional drought (Wilhite, 2000; Zhai & Feng, 2009; Zhang et al., 2012; Van Lanen et al., 2016; Santos et al., 2019). The regional monitoring of drought has significantly influenced the country’s economy and other human activities (Zhai & Feng, 2009; Mousavikhah, Shayegh & Ahmadpari, 2020). The regional drought monitoring highlights those issues if they could improve at the regional level before the events occur, then potential adverse effects of drought can be minimized in the future (Wilhite, 2000; Santos et al., 2019; Pontes Filho et al., 2019; Pontes Filho et al., 2020). More comprehensive and accurate drought monitoring can be possible by applying suitable tools and techniques according to climatic conditions. Based on the various climatic conditions, several drought indices have been used for drought monitoring. These indices require proper and effective recording related to drought occurrences. For instance, more accurate estimation of drought indices requires appropriate gauge stations with suitable records for regional drought.

Information obtained from the drought indicators can be used for improving drought predicting and forecasting (Tsakiris, Pangalou & Vangelis, 2007; Niemeyer, 2008; Hayes et al., 2011; Mukherjee, Mishra & Trenberth, 2018). Moreover, various studies from the literature have discussed the drought indices. Several studies have developed some new drought indices (McKee, Doesken & Kleist, 1993; Tsakiris, Pangalou & Vangelis, 2007; Vicente-Serrano, Beguería & López-Moreno, 2010). The development in drought monitoring is leading to enhancing the capabilities of drought monitoring more precisely and accurately. The standardized indices are used for the drought classifications. The estimation of the drought indices is based on the various parameters (precipitation, temperature, etc.). However, the preference for calculating the indices is based on climatic conditions of the available data (Niemeyer, 2008; Hayes et al., 2011). For instance, an index based on the precipitation, which is known as Standardized Precipitation Index (SPI) proposed by McKee, Doesken & Kleist, 1993, the Reconnaissance Drought Index (RDI) developed by Tsakiris, Pangalou & Vangelis (2007), the Vicente-Serrano, Beguería & López-Moreno (2010) has proposed an index which is called as Standardized Precipitation Evapotranspiration Index (SPEI).

Further, knowledge about the spatiotemporal characteristics of drought occurrences is crucial for drought monitoring and mitigation policies (Maybank et al., 1995; SIRDAŞ & Sen, 2003; Wang et al., 2020; Zhou et al., 2020). Specifically, in the presence of a homogenous region, the indicators need comprehensive characterization of the drought that provides regional spatiotemporal information. Information obtained from the spatiotemporal characteristics can be used for significant, drought monitoring, modeling, and prediction (Maybank et al., 1995; SIRDAŞ & Sen, 2003; Corzo Perez et al., 2011; Wang et al., 2020; Diaz et al., 2020b). Therefore, an intense spatiotemporal procedure is required to assimilate the spatiotemporal information of the selected homogenous region (Dabanlı, Mishra & Şen, 2017; Caloiero et al., 2018; Zhou et al., 2020). In this regard, we aimed to develop a new drought assessment procedure for the regional drought characterization. The proposed procedure is known as the Maximum Spatio-Temporal Two-Stage Standardized Weighted Index (MSTTSSWI). The MSTTSSWI is based on the Spatio-Temporal Two-Stage Standardized Weighting Scheme (STTSSWS) and validated to the six meteorological stations of the northern area of Pakistan. The proposed MSTTSSWI provides more comprehensive and accurate information about the regional drought characteristics.

## Methods

### Standardized drought index

The various Standardized Drought Indices (SDI) have been used to monitor drought (Alley, 1984; Narasimhan & Srinivasan, 2005; Stagge et al., 2015; Eslamian et al., 2017; Niaz et al., 2020). However, the SPI has been commonly used for drought assessment and can be calculated at various time scales. Several studies have used SPI for drought monitoring (Stagge et al., 2015; Eslamian et al., 2017; Pathak & Dodamani, 2019; Hagenlocher et al., 2019; Niaz et al., 2020). Further, the SPEI is a multi-scalar drought index that attracted significant attraction in drought estimation. The SPEI was developed by Vicente-Serrano, Beguería & López-Moreno (2010) that obtains the simplicity in temporal characterization and considers as an extension of SPI. SPEI evaluates the effects of evaporative demand on drought and is computed by considering both precipitation and potential evapotranspiration. More detailed information concerning the SPEI computation can be acquired in Vicente-Serrano, Beguería & López-Moreno (2010) and Beguería et al. (2014). Further, both SDI (SPI and SPEI) are frequently used in various studies to assess drought in various regions. The calculation and data availability are relatively easy; therefore, these two indices are commonly used worldwide. Hence, based on the availability of the data, the current study considers both SDI (SPI, and SPEI) for the current analysis. Both SDI requires the appropriate transformation method for standardizing the selected cumulative density function and all the numerical vectors containing the time series data based on the probability plotting formulas. Therefore, following the same procedure is used as a transformation method for standardizing SPI and SPEI (Farahmand & AghaKouchak, 2015).

(1) $$SDI = - \left(v - {{{l_0} + {l_1}v + {l_2}{v^2}} \over {1 + {m_0}v + {m_1}{v^2} + {m_2}{v^3}}}\right)$$

For

$v = \sqrt {\ln \left[ {{1 \over {{{\left\{ {T\left( x \right)} \right\}}^2}}}} \right]}$

When

(2) $$0 \le T\left( x \right)\le 0.5$$

(3) $$SDI = + \left(v - {{{l_0} + {l_1}v + {l_2}{v^2}} \over {1 + {m_0}v + {m_1}{v^2} + {m_2}{v^3}}}\right)$$

And for

$\matrix{ {} \hfill \cr {v = \sqrt {\ln \left[ {{1 \over {{{\left\{ {T\left( x \right)} \right\}}^2}}}} \right]} } \hfill \cr }$

When

(4) $$0.5 \le T\left( x \right)\le 1$$

where ${l_0} = 2.515517$, ${l_1} = 0.802853$, ${l_2} = 0.010328$, ${m_0} = 1.432788$, ${m_1} = 0.189269$, ${m_2} = 0.001308$.

### The weighting scheme: the spatio-temporal two-stage standardized weighting scheme (STTSSWS)

Drought causes severe damages worldwide. However, drought monitoring policies need a deep knowledge regarding the spatial and temporal distribution of drought risk at the local or regional level. Therefore, in this perspective, we propose STTSSWS, the innovative methodology giving a better evaluation and management of drought monitoring, especially for spatial and temporal characteristics of the region. The STTSSWS is based on steady-state probabilities. The steady-state probabilities can be defined as the average probability that the system remains in a certain state after many transitions.

Moreover, in a Markov process, it can be more explicitly defined as the probabilities approach the steady-state probabilities after some periods have been passed. Further, detailed mathematical explanations related to the steady-state probabilities of the Markov chain are presented in Stewart (2009). Moreover, in STTSSWS, steady-state probabilities are used as weights in two stages. The application of steady-state probabilities is available (Niaz et al., 2020). The Niaz et al. (2020) used the steady-state probabilities as a weighting scheme for their studies. They obtained steady states weights from the long run time series data for various drought categories in the selected region. The steady-state probabilities for the drought categories are defined as the visit of the specific drought category in the long run. The steady states probabilities for various drought categories with their probabilities, ${w'_{ij}}$, ${w''_{ij}}$ are given as follows:

$\rm {{\hskip12.6pc}ED\quad \ SD\quad \ MD\ \quad ND\quad MW\quad SW\quad EW}$

$\rm {Steady{-} state\; probabilities\; for\; SPI} =[\matrix{ {{{w'}_{11}}} \quad {{{w'}_{21}}} \quad {{{w'}_{31}} \quad {{w'}_{41}} \quad {{w'}_{51}} \quad {{w'}_{61}} \quad {{w'}_{71}}} \cr } ]$

${\rm {\hskip13pc}EW \ \quad SW \ \quad MW \quad ND \quad \ MD\quad \ SD \ \quad ED}$

${\rm Steady{-}state\; probabilities\; for\; SPEI }= [\matrix{ {{{w''}_{11}}} \quad {{{w''}_{21}}} \quad {{{w''}_{31}}\quad {{w''}_{41}}\quad {{w''}_{51}}\quad {{w''}_{61}}\quad {{w''}_{71}}} \cr } ]$

Further, the limiting probability in each index for varying drought categories is a 1 × 7-row vector given by the following expressions.

(5) $$\eqalign{
  & \prod\limits_i {(SPI)}  = [\prod\limits_1 {(E{D_{SPI}})} \prod\limits_2 {(S{D_{SPI}})} \prod\limits_3 {(M{D_{SPI}})} \prod\limits_4 {(N{D_{SPI}})} \prod\limits_5 {(M{W_{SPI}})}   \cr 
  & \quad \quad \quad \quad \prod\limits_6 {(S{W_{SPI}})} \prod\limits_7 {(E{W_{SPI}})} ] \cr} $$

(6) $$\eqalign{
  & \prod\limits_i {(SPEI)}  = [\prod\limits_1 {(E{D_{SPEI}})} \prod\limits_2 {(S{D_{SPEI}})} \prod\limits_3 {(M{D_{SPEI}})} \prod\limits_4 {(N{D_{SPEI}})} \prod\limits_5 {(M{W_{SPEI}})}   \cr 
  & \quad \quad \quad \quad \quad \prod\limits_6 {(S{W_{SPEI}})} \prod\limits_7 {(E{W_{SPEI}})} ] \cr} $$

Equations (5) and (6) give the long-run probabilities for each drought category for SPI and SPEI, and these probabilities are considered as initial weights for the calculation of STTSSWS. The calculation of STTSSWS is based on two stages. In the first stage, steady states probabilities were used to obtain weights corresponding to each drought category of every month of the time-series data. The monthly time series data are collected from January 1971 to December 2017. Thus, each month appears with a specific drought category. The drought categories are specified by the thresholds values of the drought indices. Further, the SPI and SPEI used the same thresholds values for the characterization of drought. For example, Normal Dry (ND) appears in any month the threshold for ND is “SDI > −1 & SDI <= 1” for both SDI (SPI and SPEI). The thresholds for other drought categories are defined accordingly. These thresholds for various drought categories are given in intervals. Hence, ND can occur in any month with a specific value of the specified interval. However, we need the particular value (not the interval) as a weight of the ND for any month in the time series data. This need becomes the reason to calculate steady-state probabilities as an initial weighting scheme. The steady-state assign the single probability for ND in the whole data set. This probability is considered as the initial weight for ND. Accordingly, the weights for other drought categories are assigned.

Furthermore, in the second stage of STTSSWS, the weights computed from steady-state probabilities for varying drought categories are used to calculate new spatiotemporal weights. The second stage of STTSSWS is divided into two phases; in the first phase, the weights obtained from steady-state probabilities are being associated with temporal characteristics of the data. For this purpose, firstly, the data of each month from the selected period is combined separately and calculated weights for each month of the year. For example, the ND appears in January 1971 at any station for selected time series data from January 1971 to December 2017; the weight for ND can be calculated using Steady-State Probability (SSP) as initial weights as follows,

(7) $$\eqalign{ Temporal\;weigh{t_{(( {ND} )\ Janurary\;1971)}} \cr= {{SS{P_{(ND)}}\;at\;Januar{y_{( {1971} )}}} \over {sum\;of\;SSP\;of\;all\;drought\;categories\;in\;all\;january\;of\;selected\;time\;series\;data}} } $$

In Eq. (7) the nominator contains SSP for only ND that is observed in January 1971, however, the denominator term consists of drought categories that appeared in all January (January of 1971, January of 1972, January of 1973, and so on till January of 2017), and their collective sum is computed. Further, we have monthly data of 47 years used at a 1-month time scale (47 * 12 = 564 months), so 47 values of January are included in the data. Accordingly, other months appear 47 times in the selected data set. The formulas are provided only for January of Skardu station to avoid the complication of the mathematical equalities. Further, several drought categories can be observed in 47 years of time series data in Skardu station (“as Eq. (6) presented only for one drought category”). Therefore, a general form is required that defines temporal weights more comprehensively. Equation (8) is provided for the calculation of January at Skardu station. However, the temporal weights for other months in other stations can be calculated based on the same rationale.

(8) $${T_{January}}({P_{(mi)(Skardu)}}) = {{{W_{(mi)Skardu}}} \over {\sum\nolimits_{i = 1}^n {{W_{(mi)Skardu}}} }},\;\;i = 1,2,3, \ldots \ldots ..,47 \;{\rm and}\;m = 1,2, \ldots ,6$$

where ${T_{January}}({P_{(mi)(Skardu)}})$ indicates the probabilities (the temporal weights) for varying drought categories in January of Skardu station. The $i$ shows the specific month (say, “January of 1971, January of 1972, January of 1973 and so on till January of 2017”) varying over the selected data set (from January 1971 to December 2017). And $m$ denotes the drought categories that are selected for the analysis (say, $m$ = (“1 (Extremely Dry (ED)), 2 (Severely Dry (SD)), 3 (Median dry (MD)), 4 (Normal Dry (ND)), 5 (Median Wet (MW)), 6 (Severely Wet (MW)), and 7 (extremely Wet (EW))”). The varying drought categories are described in Niaz et al. (2020). The steady-state weights for various drought categories for January of any year at the Skardu station are given by ${W_{(mi)Skardu}}$. The $\sum\nolimits_{i = 1}^n {{W_{(mi)Skardu}}}$ shows that the steady-state weights are added for all January at Skardu station with several drought categories. Moreover, $n$ show the total months of January (*i.e*., 47) in Skardu. For instance, the nominator term ${W_{(mi)Skardu}}$ is computed with several drought categories for Skardu station for the various months of January, and the denominator term contains drought categories that appeared in all January at Skardu station. Now, the monthly weights for other months (February, up to December) with these selected categories are evaluated on the same rationale. Furthermore, the second phase perceives spatiotemporal characteristics of the selected drought categories. Thus, the spatiotemporal weights for these drought categories can be obtained as follows,

(9) $$S{T_{January}}({P_{\left( {mi} \right)\left( {Skardu} \right)})}={{{T_{January}}({P_{\left( {mi} \right)\left( {Skardu} \right)}})} \over {\sum\nolimits_{j = 1}^M {{Q_{mij}}} }},\;\;\;i = 1,2,3, \ldots ,47 \;{\rm and}\;j = 1,2,, \ldots ,6$$

where Eq. (9) takes monthly spatiotemporal weights for various drought categories at Skardu station. $S{T_{January}}({P_{\left( {mi} \right)\left( {Skardu} \right)}}$) Shows the probabilities (spatiotemporal weights) computed from spatiotemporal information for varying drought categories in January at Skardu station. Further, the weights ${T_{January}}({P_{\left( {mi} \right)\left( {Skardu} \right)}})$ which were calculated from Eq. (8), are being further divided by the $\sum\nolimits_{j = 1}^M {{Q_{mij}}}$. Here, the quantity ${Q_{mij}}$ can be obtained for varying January of the selected period by adding varying drought categories ( $m$) observed at various selected stations ( $j$) and the total number of selected stations are denoted by $M$ (*i.e.*, $M$ = 6). The STTSSWS uses spatiotemporal characteristics of the selected stations and provides more accurate information about drought occurrences in a homogenous region. The obtained information from the STTSSWS can be used to build substantial drought monitoring procedures, techniques, and methodologies.

### The proposed MSTTSSWI method for selecting regional drought characteristics

**Figure 1:**
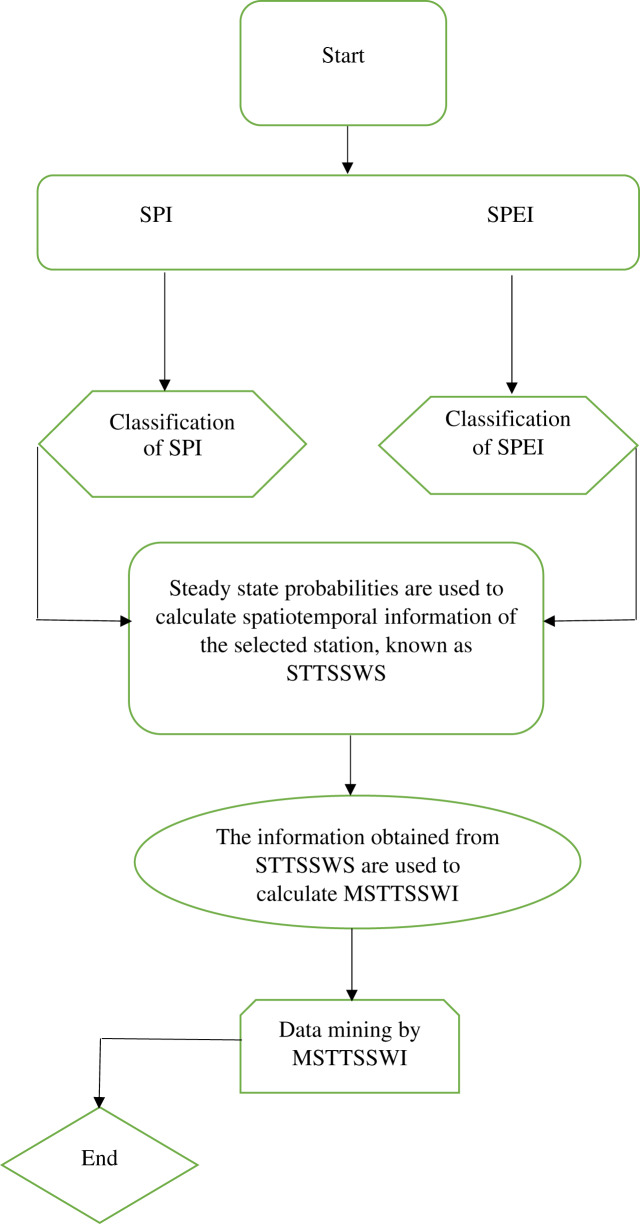
Flowchart. Flowchart for the proposed MSTTSSWI.

The six homogenous stations are selected for the validation of the proposed procedures. The STTSSWS assigns weights to the various drought categories in each station. Further, the current study extends the concept of Niaz et al. (2020). The mentioned study proposed a regional drought index at a 1-month time scale that selects suitable drought categories from the various homogenous stations. They used steady-state probabilities as a weighting scheme since they are associated with temporal information and are not considered spatial characteristics. Therefore, in the current study, the STTSSWS is used to obtain spatiotemporal information of the region. The obtained information from the STTSSWS is used to calculate suitable drought categories of the region. The vector of varying drought categories for regional characterization obtained from STTSSWS is known as the Maximum Spatio-Temporal Two-Stage Standardized Weighted Index (MSTTSSWI) (Fig. 1). The mathematical form of the MSTTSSWI can be presented for SPEI at a 1-month time scale for varying stations as follows,

(10) $${}{\rm MSTTSSWI}=\left\{ {\matrix{ {SPEI\ Skardu\ if\prod\limits_i {\left( {Skardu} \right)} \gt \prod\limits_i {\left( {Gilgit} \right)} \gt \prod\limits_i {\left( {Chilas} \right)} \gt \prod\limits_i {\left( {Gupis} \right)} \gt \prod\limits_i {Bunji} ) \gt \prod\limits_i {\left( {Astore} \right)} } \cr {}{SPEI\ Gilgit\ if\prod\limits_i {\left( {Gilgit} \right)} \gt \prod\limits_i {\left( {Chilas} \right)} \gt \prod\limits_i {\left( {Gupis} \right)} \gt \prod\limits_i {\left( {Bunji} \right)} \gt \prod\limits_i {\left( {Astore} \right)} } \cr {}{SPEI\ Chilas\ if\prod\limits_i {\left( {Chilas} \right)} \gt \prod\limits_i {\left( {Gupis} \right)} \gt \prod\limits_i {\left( {Bunji} \right)} \gt \prod\limits_i {\left( {Astore} \right)} } \cr {}{SPEI\ Gupuis\ if\prod\limits_i {\left( {Gupis} \right)} \gt \prod\limits_i {\left( {Bunji} \right)} \gt \prod\limits_i {\left( {Astore} \right)} } \cr {}{SPEI\ Bunji\ if\prod\limits_i {\left( {Gilgit} \right)} \gt \prod\limits_i {\left( {Astore} \right)} }\cr {}{SPEI\ Astore,otherwise} \cr } } \right.$$

The MSTTSSWI is given in Eq. (10) for six selected stations. Each station may have varying drought categories for the selected time (from January 1971 to December 2017). The STTSSWS is used to assign weights for varying drought categories and stations. However, among the weights assigned to each selected station, the stations that receive maximum weights are selected in MSTTSSWI. Therefore, the vector of MSTTSSWI contains all suitable drought categories that are observed in various stations. For example, In January 1974, using SPI at a 1-month time scale, the STTSSWS assigns weights for varying drought categories as median wet (MW) by 0.06, (MW = 0.06), (MW = 0.07), normal dry (ND = 0.38), (ND = 0.41), and severely wet (SW = 0.03) observed at Skardu, Gilgit, Chilas, Gupis, Bunji, and Astore, respectively. In this case, for January 1974, the MSTTSSWI chooses the ND = 0.41, which is the maximum weight obtained from STTSSWS among various categories and stations. Accordingly, the selection is made for suitable drought categories for other months using SDI (SPI and SPEI). Conclusively, the MSTTSSWI provides the single vector of various appropriate drought categories among the six selected stations.

## Application

**Figure 2:**
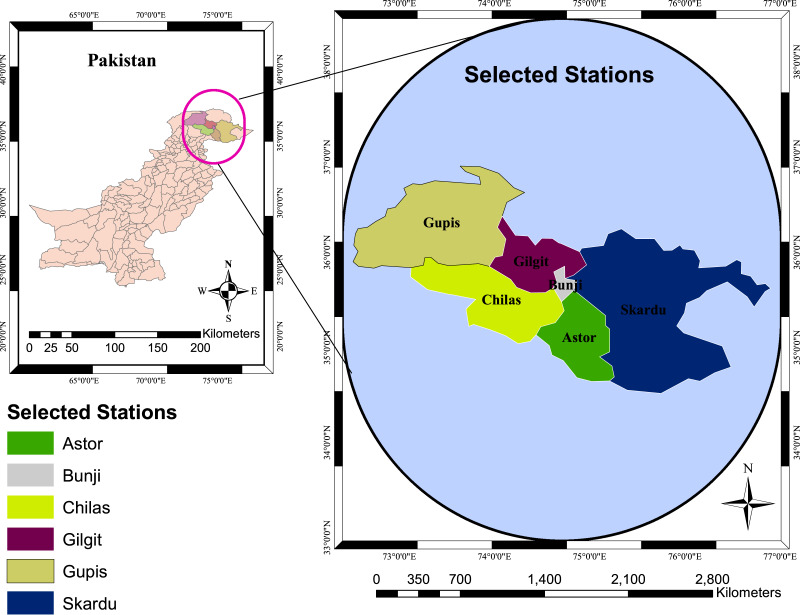
Geographical locations of the selected stations.

The six meteorological stations of the northern areas of Pakistan (Fig. 2) are selected in STTSSWS for the regional drought analysis. Northern Area is a geographic area with three mountain ranges, the Himalayas, Karakoram, and the Hindu Kush, which cover most of the region (Rasul et al., 2011). Many of the world’s tallest peaks are found in this region, including K-2, Nanga Parbat, and Rakaposhi. The average altitude of Karakorum is (6,100 M), Hindukush (7,690 M) and Himalaya (8,848 M) (Latif et al., 2020). These high altitudes of mountains frequently deliver a significant portion of precipitation (Rasul et al., 2011; Bocchiola & Diolaiuti, 2013; Adnan et al., 2017). Further, this region’s precipitation and temperature have substantial effects on the country’s other regions (Anjum et al., 2010; Bocchiola & Diolaiuti, 2013; Mazhar et al., 2020; Adnan et al., 2017). Therefore, the precipitation and temperature of the selected region are used in STTSSWS to substantiate drought occurrences. In addition, the obtained information from STTSSWS is used to calculate MSTTSSWI that provides a regional characterization of meteorological drought.

### Results

**Figure 3:**
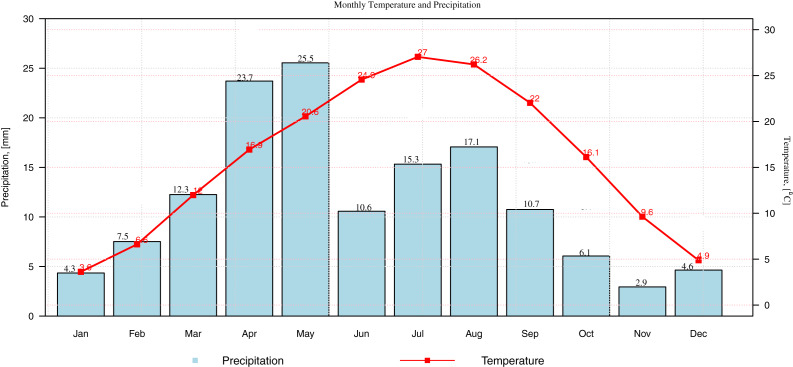
The climograph. The monthly precipitation and temperature are presented for Gilgit station.

The monthly data of precipitation, maximum and minimum temperature are observed in various stations. The observed data of these indicators (precipitation and temperature) are used for the current analysis. The monthly mean precipitation and mean monthly temperature (maximum and minimum temperature) for Gilgit station is presented in a climograph (Fig. 3). To avoid the presentation of multiple figures the data of Gilgit station is used for the climogrpah, however, the behavior of the selected indicators can be presented for other selected stations (Bunji, Gupis, Chilas, Skardu and Astore) accordingly. Further, based on the climatic conditions of the selected stations, two standardized drought indices are selected for drought classification. The standardization of these drought indices is done by using varying probability distributions. The distributions which are suitable according to climatic conditions are chosen for the standardizations. The Bayesian Information Criterion (BIC) is used to select appropriate probability distributions. In Table 1, at a one-month time scale, the 3p Weibull distribution shows suitable candidacy for the Astor station. The BIC of 3p Weibull distribution is −1036.5which is minimum among other distributions. Therefore, the distribution is used for the standardization in this station. The 3p Weibull distribution, at a 1-month time scale, shows suitable candidacy for Bunji station with BIC (−1,031.0), Gilgit with BIC (−1,097), and for Skardu with BIC (−735.1). The 4p Beta distribution shows better candidacy at a 1-month SPI for two stations, including Gupis and Chilas with BIC −788.7 and −805.6, respectively. Further, for SPEI at a 1-month time scale, the Trapezoidal distribution is fitting suitably for station Astor and Skardu with BIC −710.1 and −664.6, respectively. In Bunji, Gupis, Chilas, and Gilgit, the Johnson SB distribution is a suitable candidate concerning their minimum BIC values, accordingly. After the standardization of the data, the observed values are classified for various drought categories by using SPI and SPEI. The drought classification shows the multiple levels of drought categories (Li et al., 2015). For instance, the SDI (SPI and SPEI) value less than or equal to −2 represents the extremely dry and greater than two classified as extreme wet conditions and so forth (Table 2).

**Table 1:** The BIC of various probability distributions.

	Astore		Bunji		Gupis	
index	Distribution	BIC	Distribution	BIC	Distribution	BIC
SPI	3p Weibull	−1,036.5	3p Weibull	−1,031.0	4p Beta	−788.7
SPEI	Trapezoidal	−710.1	Johnson SB	−1,248.4	Johnson SB	−977.6

	Chilas		Gilgit		Skardu	
index	Distribution	BIC	Distribution	BIC	Distribution	BIC
SPI	4P Beta	−805.6	3P Weibull	−1,097.4	3P Weibull	−735.1
SPEI	Johnson SB	−594.7	Johnson SB	−1,213.2	Trapezoidal	−664.6

**Note:**

The BIC of various probability distributions for selected stations at scale-1 for SPI and SPEI.

**Table 2:** The preliminary classification. The preliminary classification of various drought categories based on SDI.

SDI	Major drought classes
SDI >= 2	Extremely Wet (EW)
SDI > 1.5 & SDI <= 2	Severely (SW)
SDI > 1 & SDI <= 1.5	Median Wet (MW)
SDI > −1 & SDI <= 1	Normal Dry (ND)
SDI > −1.5 1 & SDI <= −1	Median Dry (MD)
SDI > −2 & SDI <= −1.5	Severely Dry (SD)
SDI >= −2	Extremely Dry (ED)

**Figure 4:**
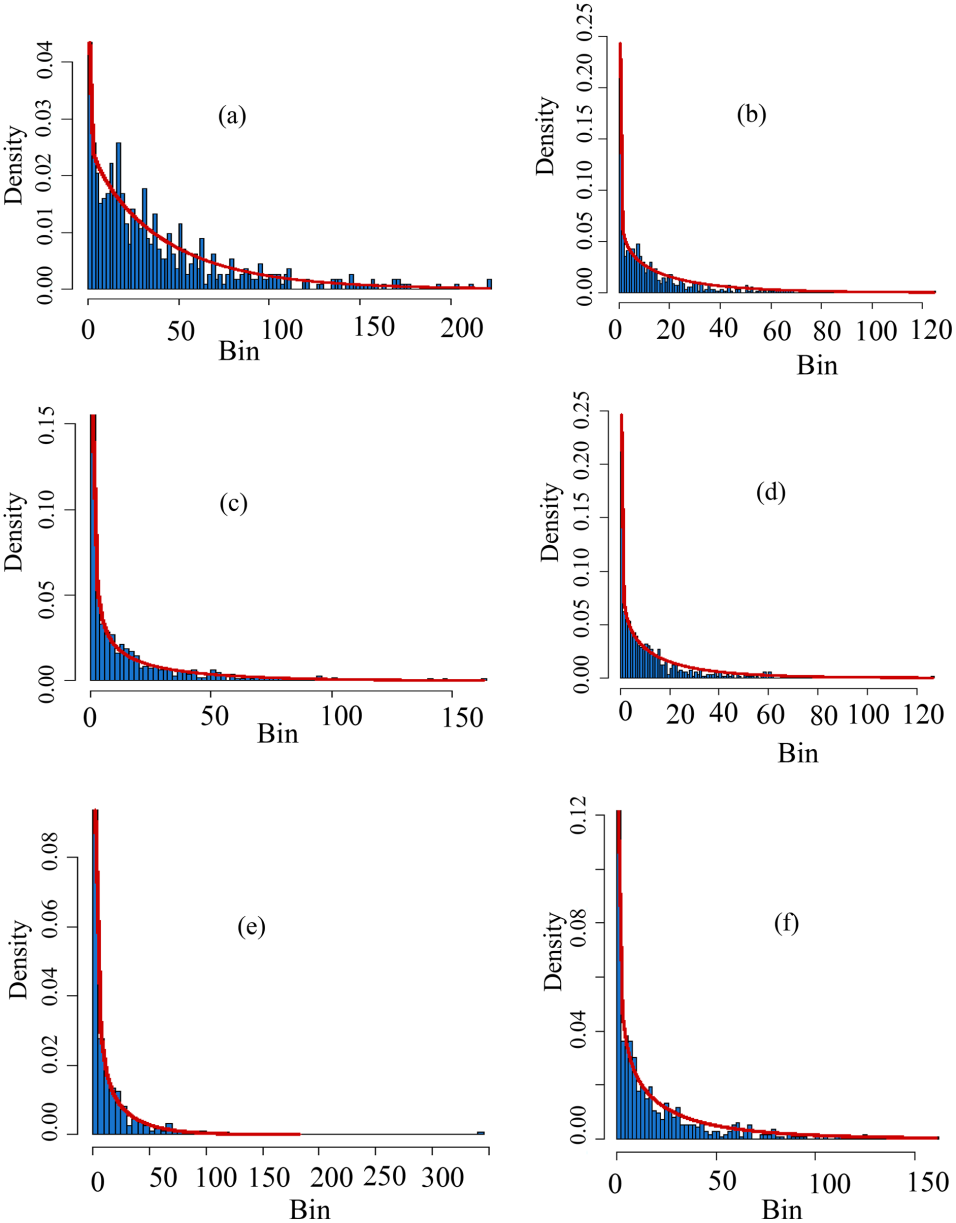
Histograms of the selected distributions. The theoretical *vs* empirical histograms are presented based on SPI at scale-1 for varying stations. For example, the theoretical *vs* empirical histograms for Astore station is presented in (A), for Bunji it is presented in (B), for Gupis it can be seen in (C), for Chilas , Giligit and Skardu, these are presented in (D–F) respectively. Further, in the multiple sections of the figure, the bins on the horizontal axis are used for ranges of data, and the ratio of the relative frequency of any specified bins’ interval to its width size is denoted by density on the vertical axis. Moreover, from the fitted lines to the multiple sections it can be observed that for SPI-1, the Gupis and Gilgit data have more closeness between the theoretical and empirical.

**Figure 5:**
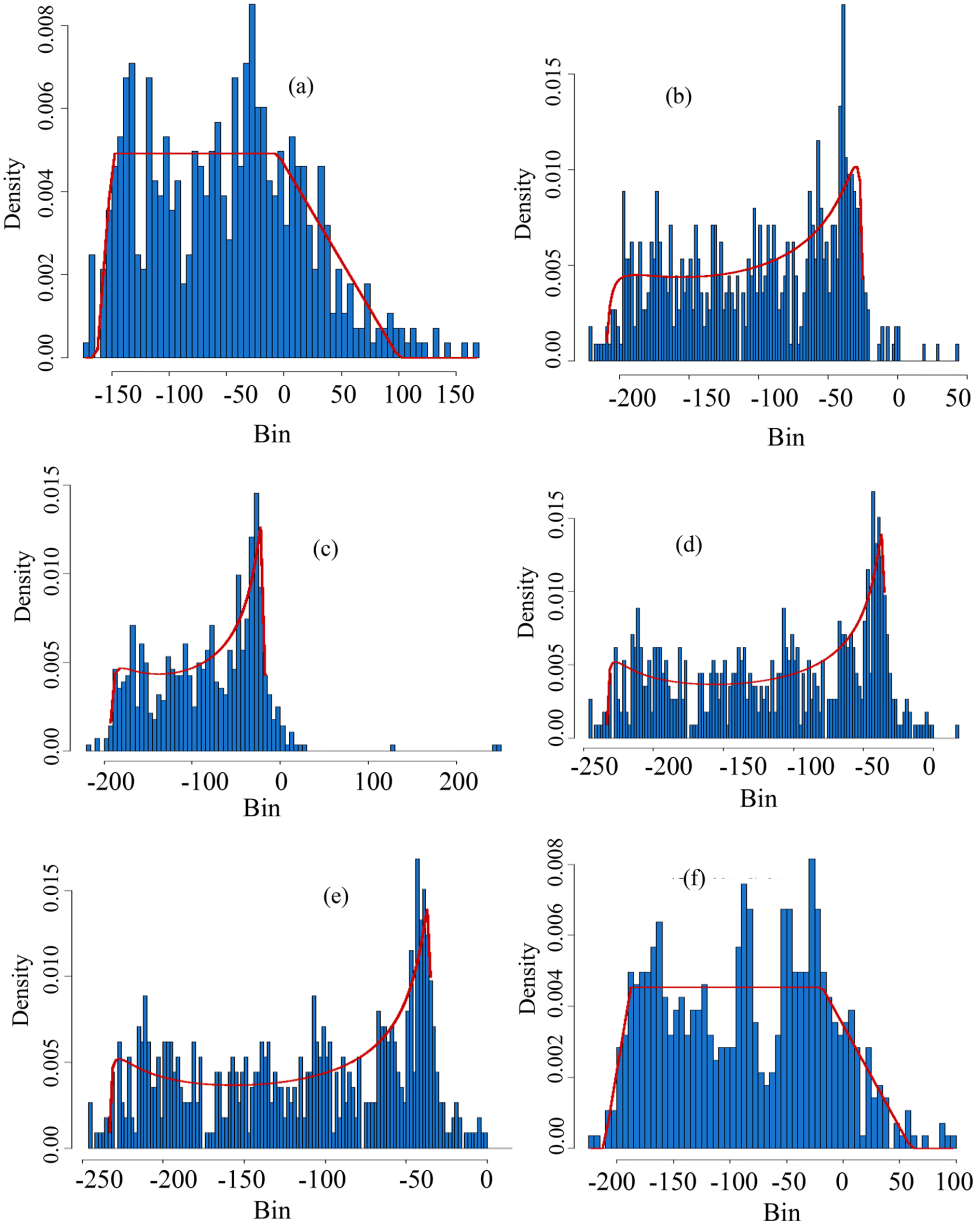
Theoretical *vs* empirical histograms. Theoretical *vs* empirical histograms of the selected distributions based on SPEI at scale-1 for selected stations are presented accordingly. The theoretical *vs* empirical histograms for Astore station is presented in (A), for Bunji it is presented in (B), for Gupis, Chilas, Giligit and Skardu, the theoretical *vs* empirical histograms are presented in (C–F), respectively.

**Figure 6:**
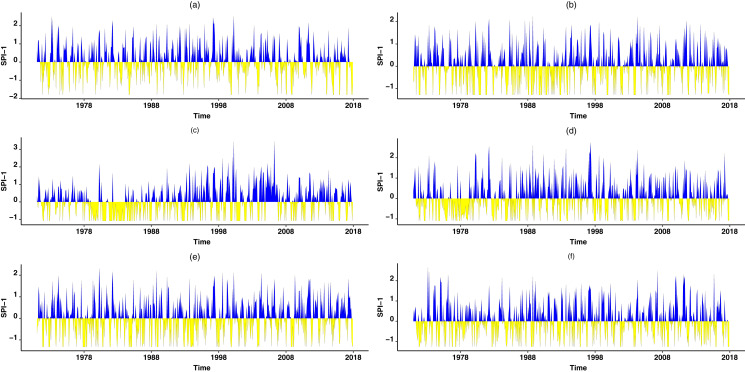
Temporal behaviour. Temporal behavior in various plots of the selected stations for SPI at scale-1 (SPI-1). The temporal behavior for SPI-1 at Astore station can be observed in (A). (B) Temporal behavior of SPI-1 in Bunji. (C) The SPI-1 temporal behavior is presented for Gupis. Moreover, the temporal behavior of SPI-1 for Chilas, Gilgit, and Skardu can be observed from (D–F), respectively.

**Figure 7:**
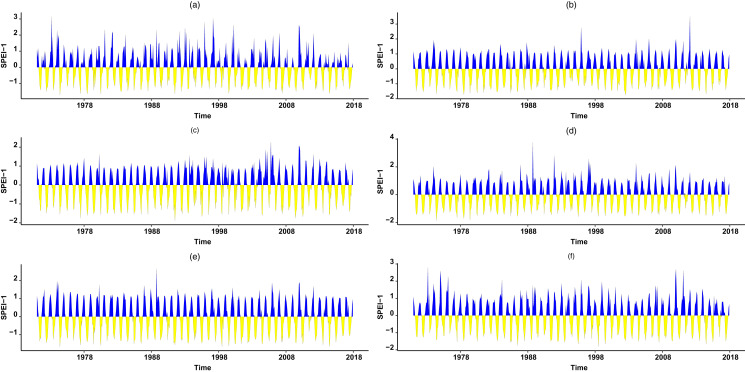
Temporal behavior in various plots for SPEI. Temporal behavior of SPEI at scale-1 (SPEI-1) can be observed in varying plots for the selected stations. The temporal behavior for SPEI-1 at Astore station can be seen in (A). (B) Temporal behavior of SPEI-1 is presented for Bunji. The temporal behavior of SPEI-1 in Gupis is presented in (C). Further, the temporal behavior of SPEI-1 for Chilas, Gilgit, and Skardu can be observed from (D–F), respectively.

**Figure 8:**
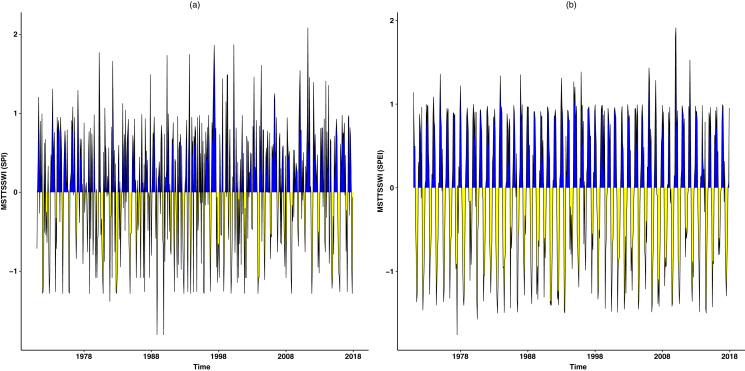
The temporal behaviour of the MSTTSSWI. The temporal behavior of the MSTTSSWI is presented based on SPI-1 and SPEI-1.

Furthermore, Fig. 4 shows theoretical and empirical distributions for SPI at a 1-month time scale (SPI-1), and theoretical and empirical distributions for SPEI at a 1-month time scale (SPEI-1) for various stations are presented in Fig. 5. Figure 6 shows the temporal behavior of SPI-1 at selected stations. Further, the temporal behavior of SPEI-1 at selected stations can be observed in Fig. 7. These indices are used to find the drought occurrences in the selected stations. The drought occurrences based on SPI and SPEI in various stations are observed accordingly. The monthly drought occurrence for the year of 2017, obtained from the SPI and SPEI are presented in Tables 3 and 4 respectively. It can be observed that in both SPI and SPEI computation the ND from the selected stations is the prevalent category. Hence, the ND category should be considered as an important category for further analysis. Further, information obtained from these indices is used for the computation of STTSSWS. The STTSSWS obtains spatiotemporal information for various stations. The obtained weights from SPI at a 1-month time scale are given in Table 5. The STTSSWS contains the temporal and spatial information of the whole region and provides more comprehensive and precise results for varying drought categories. For example, in January, for the Astore station at the 1-month time scale of SPI, the SD takes a value of 0.0268. The value shows that the SD has very less likely to occur in January. However, in January, the ND is more likely to occur in Gilgit station among other stations of the region with the weight (0.2672). For other stations and months, the weights for varying drought categories can be observed. Furthermore, STTSSWS weights for a homogenous region using SPEI at a 1-month time scale are presented in Table 6. In the Astore station, at the 1-month time scale of SPEI, the MW takes a value of 0.0836. The value shows that the MW has significantly less likely to occur in January at Gilgit. However, in January, the ND is more likely to occur in the Skardu station among other region stations with the weight (0.1844). Moreover, to avoid the complexity in presenting results, we just presented results for the particular year, 2017. However, the results of selected years can be observed from the proposed scheme. Further, the STTSSWS is used to assign weights for the selected drought categories on varying stations. The STTSSWS is applied to the six meteorological stations to obtain spatiotemporal weights for estimating MSTTSSWI. Hence, the MSTTSSWI provides more comprehensive and accurate information for the regional drought characteristics. The behavior of the proposed MSTTSSWI can be observed by (Fig. 8).

**Table 3:** The classification of the drought categories.

	Astore		Bunji		Gupis		Chilas		Gilgit		Skardu	
	Index	Classif.	Index	Classif.	Index	Classif.	Index	Classif.	Index	Classif.	Index	Classif.
January	−1.8044	SD	−1.2152	MD	−1.0805	MD	−0.3254	ND	−0.2296	ND	−0.4827	ND
February	−1.8044	SD	0.2629	ND	−1.0805	MD	0.8075	ND	0.2014	ND	−1.2581	ND
March	−0.4745	ND	−1.1059	MD	−0.6655	ND	−0.1535	ND	−1.0523	MD	−0.3096	ND
April	1.8879	SW	0.9377	ND	1.2811	MW	2.2094	EW	1.8283	SW	1.0615	EW
May	0.3314	ND	0.6975	ND	0.9813	ND	0.8297	ND	0.9724	ND	0.0796	ND
June	−0.3302	ND	−0.6380	ND	−0.2883	ND	−0.0637	ND	0.9309	ND	−0.8574	ND
July	−0.1857	ND	0.8192	ND	0.7872	ND	0.1366	ND	1.0574	MW	−0.2401	ND
August	−0.1967	ND	0.7181	ND	1.1317	MW	0.2058	ND	0.3864	ND	0.0086	ND
September	−0.5938	ND	0.2824	ND	0.4029	ND	0.1937	ND	0.4759	ND	0.0739	ND
October	−1.7685	SD	−1.1576	MD	−0.2883	ND	−1.0539	MD	−0.9569	ND	−1.2581	MD
November	−1.8044	SD	−1.2806	MD	−1.0805	MD	−1.0995	MD	−1.3227	MD	−1.2581	MD
December	−1.2499	MD	−1.2806	MD	−0.0621	ND	−1.0539	MD	−1.2398	MD	−1.2168	MD

**Note:**

The classified (Classif.) based on SPI. The varying drought categories observed in various months of the year 2017, in selected stations.

**Table 4:** The classification based on SPEI.

	Astore		Bunji		Gupis		Chilas		Gilgit		Skardu	
	Index	Classif.	Index	Classif.	Index	Classif.	Index	Classif.	Index	Classif.	Index	Classif.
January	0.2741	ND	0.9796	ND	0.7679	ND	0.9039	ND	1.0670	MW	0.8220	ND
February	0.0829	ND	0.8384	ND	0.4871	ND	0.9296	ND	0.7976	ND	0.4919	ND
March	−0.1682	ND	0.1045	ND	0.0219	ND	0.1193	ND	0.1447	ND	0.1232	ND
April	1.4903	MW	0.0887	ND	0.3163	ND	1.3240	MW	0.6436	ND	0.1957	ND
May	−0.8214	ND	−1.0027	MD	−0.7708	ND	−0.8617	ND	−0.7646	ND	−0.9650	ND
June	−1.3547	MD	−1.3982	MD	−1.4032	MD	−1.3619	MD	−1.0659	MD	−1.4873	MD
July	−1.2755	MD	−1.0733	MD	−1.2931	MD	−1.3665	MD	−1.0312	MD	−1.3089	MD
August	−0.9560	ND	−0.7027	ND	−0.4335	ND	−0.8499	ND	−0.6704	ND	−0.8895	ND
September	−0.7888	ND	−0.4535	ND	−0.5996	ND	−0.4873	ND	−0.3957	ND	−0.4748	ND
October	−0.4409	ND	0.0387	ND	−0.0183	ND	0.0061	ND	0.0157	ND	−0.0880	ND
November	0.0596	ND	0.7154	ND	0.5016	ND	0.6265	ND	0.6878	ND	0.4532	ND
December	0.2411	ND	0.9475	ND	0.8261	ND	0.8367	ND	0.9538	ND	0.6461	ND

**Note:**

The classified (Classif.) drought categories observed in various months for SPEI at a 1 month-time scale of the year 2017, in selected stations.

**Table 5:** The weights obtained from STTSSWS.

SPI-1													
Station	Astore		Bunji		Gupis		Chilas		Gilgit		Skardu		
Month	Category	Weight	Category	Weight	Category	Weight	Category	Weight	Category	Weight	Category	Weight	Sum
January	SD	0.0268	MD	0.0750	MD	0.1106	ND	0.2566	ND	0.2672	ND	0.2638	1
February	SD	0.0233	ND	0.2126	MD	0.1068	ND	0.2175	ND	0.2179	ND	0.2219	1
March	ND	0.2397	MD	0.0545	ND	0.1898	ND	0.2295	MD	0.0541	ND	0.2325	1
April	SW	0.0606	ND	0.7149	MW	0.1037	EW	0.0333	SW	0.0582	EW	0.0294	1
May	ND	0.1646	ND	0.1769	ND	0.1610	ND	0.1571	ND	0.1817	ND	0.1587	1
June	ND	0.1609	ND	0.1637	ND	0.1959	ND	0.1626	ND	0.1518	ND	0.1653	1
July	ND	0.1789	ND	0.2072	ND	0.1978	ND	0.1948	MW	0.0241	ND	0.1971	1
August	ND	0.1794	ND	0.2221	MW	0.0314	ND	0.1823	ND	0.2014	ND	0.1833	1
September	ND	0.1815	ND	0.1848	ND	0.1624	ND	0.1554	ND	0.1592	ND	0.1568	1
October	SD	0.0372	MD	0.1053	ND	0.3256	MD	0.1074	ND	0.3396	MD	0.0849	1
November	SD	0.1815	MD	0.1848	MD	0.1624	MD	0.1554	MD	0.1592	MD	0.1568	1
December	MD	0.0600	MD	0.1342	ND	0.3846	MD	0.1511	MD	0.1481	MD	0.1221	1

**Note:**

The weights obtained from STTSSWS are provided for the year 2017. These weights are calculated by SPI and steady-state probabilities.

**Table 6:** The weights obtained from STTSSWS are given for the year 2017 using SPEI and steady-state probabilities.

SPEI-1													
Station	Astore		Bunji		Gupis		Chilas		Gilgit		Skardu		
Month	Category	Weight	Category	Weight	Category	Weight	Category	Weight	Category	Weight	Category	Weight	Sum
January	ND	0.1379	ND	0.2569	ND	0.1697	ND	0.1676	MW	0.0836	ND	0.1844	1
February	ND	0.1672	ND	0.1651	ND	0.1391	ND	0.1771	ND	0.1661	ND	0.1854	1
March	ND	0.2147	ND	0.1435	ND	0.1471	ND	0.1637	ND	0.1411	ND	0.1898	1
April	MW	0.0359	ND	0.2276	ND	0.2348	MW	0.0401	ND	0.2322	ND	0.2295	1
May	ND	0.2056	MD	0.0616	ND	0.1878	ND	0.1900	ND	0.1768	ND	0.1781	1
June	MD	0.1146	MD	0.1925	MD	0.1429	MD	0.1899	MD	0.1952	MD	0.1649	1
July	MD	0.1509	MD	0.1359	MD	0.1898	MD	0.1668	MD	0.1827	MD	0.1739	1
August	ND	0.1688	ND	0.1619	ND	0.1693	ND	0.1525	ND	0.1610	ND	0.1866	1
September	ND	0.1852	ND	0.1815	ND	0.0816	ND	0.1853	ND	0.1815	ND	0.1848	1
October	ND	0.1644	ND	0.1645	ND	0.1645	ND	0.1644	ND	0.1779	ND	0.1643	1
November	ND	0.1720	ND	0.1642	ND	0.1618	ND	0.1719	ND	0.1655	ND	0.1647	1
December	ND	0.1087	ND	0.2373	ND	0.1276	ND	0.1371	ND	0.2683	ND	0.1211	1

**Note:**

The weights obtained from STTSSWS are given for the year 2017. These weights are calculated by SPEI and steady-state probabilities accordingly.

### Discussion

The two drought indices (SPI & SPEI) are considered in the current analysis. These selected drought indices provide the standardized values for the given climate indicators (precipitation and temperature) in the selected stations. The appropriate probability distributions according to time scales and stations are selected for the standardization (Niaz et al., 2020; Ali et al., 2020; Niaz et al., 2021; Raza et al., 2021). The BIC criteria are used to select these probability distributions. Further, the steady-state probabilities are used for the computation of STTSSWS. The STTSSWS works in two stages; in the first stage, SPI, SPEI, and the steady-state probabilities are calculated for each station separately. The steady-state probabilities consider temporal information of the stations regardless of the spatial accountability of the region. However, the current study aimed to improve the information of the regional drought characteristics. Therefore, in stage two of the STTSSWS, the steady-state probabilities are used to calculate spatiotemporal information (weights) for the varying drought categories. Hence, the proposed scheme used spatial and temporal characteristics of regional drought to calculate weights for the various drought categories. Various studies emphasized calculating spatiotemporal information of the drought (Corzo Perez et al., 2011; Wang et al., 2020; Diaz et al., 2020a). The knowledge about spatiotemporal characteristics of the drought can help for accurate drought monitoring characteristics and can be used for significant modeling and drought prediction (Caloiero et al., 2018; Zhou et al., 2020). Therefore, the current study aimed to develop a new drought assessment procedure for the characterization of regional drought. The MSTTSSWI uses STTSSWS as a weighing scheme to provide more comprehensive and accurate information about the regional drought characteristics.

## Conclusion

The current study proposes a new drought assessment procedure, known as MSTTSSWI based on STTSSWS. The SPI, SPEI, and steady-state probabilities are used in STTSSWS to obtain new spatiotemporal weights for various drought categories. Further, the STTSSWS is used as a weighting scheme to calculate MSTTSSWI to obtain more accurate and precise spatiotemporal information about drought occurrences at the regional level. The outcomes of the proposed procedure MSTTSSWI provide regional spatiotemporal characteristics for the drought in the selected region and motivate researchers and policymakers to use the more comprehensive and accurate spatiotemporal characterization of drought in the selected region. Information obtained from MSTTSSWI can be applied for monitoring and forecasting drought more accurately. Moreover, when the climatic conditions of the stations change, the proposed MSTTSSWI works accordingly to the specific conditions.

## Supplemental Information

10.7717/peerj.13249/supp-1 Supplemental Information 1 The dataset that calculated for SPI on various stations and steady states weights. The steady states weights are included.

10.7717/peerj.13249/supp-2 Supplemental Information 2 The calculated data from SPEI and steady state weights for each drought category in various stations.

10.7717/peerj.13249/supp-3 Supplemental Information 3 Classification example for Bunji station at SPI scale1 is provided.

10.7717/peerj.13249/supp-4 Supplemental Information 4 Raw data of precipitation for selected stations.

## Additional Information and Declarations

### Data Availability

The following information was supplied regarding data availability: The data is available in the Supplemental File.
